# Prevalence and risk factors of self-reported hearing loss, tinnitus, and dizziness in a population-based sample from rural northeastern Germany

**DOI:** 10.1038/s41598-024-68577-3

**Published:** 2024-07-31

**Authors:** Friedrich Ihler, Tina Brzoska, Reyhan Altindal, Oliver Dziemba, Henry Völzke, Chia-Jung Busch, Till Ittermann

**Affiliations:** 1 Department of Otorhinolaryngology, Head and Neck Surgery, Greifswald University Medicine, Fleischmannstrasse 8, 17475 Greifswald, Germany; 2 Institute for Community Medicine, University Medicine Greifswald, 17475 Greifswald, Germany

**Keywords:** Risk factors, Auditory system, Inner ear, Sensory processing

## Abstract

A close anatomical and physiological relationship is known between the senses of hearing and balance, while an additional pathophysiological interaction is supposed. The mechanisms underlying this association are not yet fully understood, especially in individuals without a known specific otologic disorder. In particular, only scarce information on the combined occurrence of audiovestibular sensory impairment is available so far. Therefore, this study aims to provide further insight into the prevalence and co-prevalence of the audiovestibular symptoms hearing loss, tinnitus and dizziness. Additionally, the influence of potential risk factors from lifestyle habits as well as cardiovascular and metabolic conditions on the development of those symptoms is studied. Data was analyzed from 8134 individuals from the population-based Study of Health in Pomerania (SHIP). SHIP pursues a broad and comprehensive examination program in chronologically separated cohorts with longitudinal follow-up. Cohorts are sampled from Western Pomerania, a rural region of north-eastern Germany. The study population represents a cross-sectional analysis from the cohorts SHIP-START (recruited 1997–2001) and SHIP-TREND (recruited 2008–2012), sampled for baseline investigations (SHIP-START-0 and SHIP-TREND-0) at the age of 20–79 years. Audiovestibular symptoms as outcome variables were assessed by structured questionnaires. Additionally, individuals were comprehensively characterized regarding modifiable lifestyle factors as well as cardiovascular and metabolic disorders, allowing the assessment of their role as exposure variables. We calculated a weighted prevalence of 14.2% for hearing loss, 9.7% for tinnitus, and 13.5% for dizziness in the population. Prevalence increased with age and differed among the sexes. A considerable share of 28.0% of the investigated individuals reported more than one symptom at once. The prevalence of hearing loss as well as tinnitus increased between the two cohorts. A moderate positive correlation was found between the occurrence of hearing loss and tinnitus (phi-coefficient 0.318). In multivariable regression analyses, education was identified as a significant protective factor while only smoking was significantly associated with all three symptoms. Furthermore, several cardiovascular risk factors contributed to both hearing loss and dizziness. In conclusion, audiovestibular symptoms are highly prevalent in the investigated population. A considerable but complex influence of risk factors points towards a relation with neuronal as well as cardiovascular disease processes. To clarify the underlying mechanisms, the interaction between the senses of hearing and balance as well as the mode of action of the risk factors should be evaluated in more detail in the future.

## Introduction

The inner ear houses both the auditory sensory organ and the vestibular labyrinth, thereby contributing to hearing and balance. Hearing loss and tinnitus are symptoms for dysfunction of the auditory system while dizziness is a general term for the subjective experience during balance disturbance. Those symptoms are considerably prevalent with hearing loss being estimated to affect up to 19.3%^1^, tinnitus up to 42.7%^2^ and dizziness up to 30%^3^ of the global population.

Variable combinations of the three symptoms can occasionally be caused by heterogenous disorders of the cerebellopontine angle or the temporal bone, including vestibular schwannoma^4^, Menière's disease^5^ or superior semicircular canal dehiscence syndrome^6^. Beyond those defined conditions, considerable evidence points towards a relevant association and co-prevalence of audiovestibular symptoms. While hearing loss is the major manifestation of auditory dysfunction, it is closely related to tinnitus. Tinnitus may be an early sign of cochlear failure that might initially be out of the detection range of common auditory tests^7^. During long-term follow-up, initially normal-hearing individuals with tinnitus progress to hearing loss in a large share of cases^8^. Vice versa, hearing loss is seen as a major risk factor for tinnitus^9,10^ and accordingly, the rehabilitation of hearing loss can provide relieve from tinnitus symptoms^10,11^. From the observation that hearing loss may have a detrimental effect on balance control^17^, an interplay between hearing and balance is supposed. This is substantiated further as the hearing system recognizes spatial information^12–14^. Additionally, auditory cues are processed during the perception of balance^14,15^ and are therefore likely to play a role in postural stability^16^. Finally, a beneficial effect on posture and gait has been reported for hearing rehabilitation^13,14,18,19^. Finally, a possible link between dizziness and tinnitus may be provided by anxiety as well as phobic, somatoform and affective disorders, since they are prevalent both in individuals with organic and non-organic disorders of the vestibular system^20^ as well as in those suffering from tinnitus^9,10^.

Regarding shared pathomechanisms, age is a major determinant of both auditory and vestibular dysfunction. Inflammatory aging processes, oxidative stress, and hereditary factors together contribute to increased prevalence with advancing age^21^. While the concept of presbycusis is well-accepted^22,23^, a supposed effect of ageing on vestibular dysfunction is less clear^24^. Multiple lifestyle factors, including low dietary quality^25–29^, physical inactivity^30–32^, alcohol consumption, obesity^9,33^, and smoking have been associated with auditory as well as vestibular dysfunction. So far, only smoking was identified as a shared risk factor of all three audiovestibular symptoms hearing loss^30,32,34–38^, tinnitus^9,33,39^, and dizziness^40,41^ and might therefore be of particular importance. Traditionally, however, hearing loss, tinnitus and dizziness were investigated separately. Many of of the named factors, in particular smoking, are also major cardiovascular risk factors. This is interesting since the presence of a cardiovascular risk profile was associated with hearing loss at the baseline investigation in a cohort study of 6974 subjects. Moreover, normal-hearing individuals at baseline had an increased risk for developing hearing loss during a 10-year follow-up when a cardiovascular risk profile was present^32^. Again for hearing loss, a link to cardiovascular events is also assumed in the other direction: individuals with sensorineural hearing loss have an increased risk of stroke, both for the distinct mechanisms of sudden sensorineural hearing loss and age-related hearing loss in a meta-analysis of eight cohort studies^42^. For dizziness and tinnitus, a comparable association is less well investigated.

Given this interconnected nature of hearing and balance, a more comprehensive analysis of the prevalence of and risk factors for individual audiovestibular symptoms is needed. Therefore, this study analyzed the prevalence of self-reported hearing loss, dizziness and tinnitus in the baseline examinations of the SHIP-START and SHIP-TREND cohorts as well as the combined occurrence of these three symptoms. In addition, it was assessed whether their occurrence is associated with age, sex, education, cardiovascular or metabolic risk factors or lifestyle habits.

## Methods

### Declarations

The Study of Health in Pomerania (SHIP) was conducted in accordance with the Declaration of Helsinki. The study protocols were approved by the responsible local ethics committee at the University Medicine Greifswald, Germany (approvals from July 31, 1995 (SHIP-START) and June 06, 2008 (SHIP-TREND, reference number BB 39/08)). All participants gave written informed consent. Ethical approval included all aspects of the present analysis, covering the structured questionnaires that contained the primary outcome variables as well as the examination program leading to the exposure variables.

### Study design and methodology

#### Study setting and general aims

SHIP is a population-based cohort study at the University Medicine Greifswald. It aims to assess prevalence and incidence of common risk factors, subclinical disorders and clinical diseases as well as to investigate the complex associations among them. Therefore, SHIP is not focused on a specific disease or organ system, but instead attempts to describe health-related conditions with the widest focus possible. Important medical areas of investigation include cardiovascular diseases, diabetes mellitus, liver and biliary tract diseases, neurological diseases, thyroid diseases, dental diseases, lung diseases, addiction and risk behavior.

Methods include self-report paper–pencil questionnaires, computer-assisted personal interviews as well as a comprehensive interdisciplinary examination program (including whole-body MRI beginning with SHIP-TREND) and the collection of biomaterials in each examination wave. The study design and protocols have been described in detail elsewhere^43,44^. While not being an explicit selection criterion, the entire sample is predominantly Caucasian due to the characteristics of the underlying population.

#### Cohorts, sampling and response

SHIP consists of independent cohorts sampled at different timepoints. The first two cohorts are named SHIP-START and SHIP-TREND, respectively. Those two cohorts finished baseline investigations in the past and already underwent one or more follow-up investigations. A third cohort (SHIP-NEXT) is currently in the process of sampling and baseline investigations, the data is not yet available. All cohorts are sampled from defined parts of mainland Western Pomerania (Vorpommern), a rural area in the northeastern German state of Mecklenburg-Vorpommern. The cohorts do not overlap, as participation in a previous cohort is an exclusion criterion for each subsequent cohort.

During initial sampling, adults aged 20–79 were selected from registration data in a randomized, two-stage cluster method stratified by sex and age^45^. The first cohort, SHIP-START, was recruited with a response rate of 68.8% while the second cohort, SHIP-TREND, showed a response rate of 50.1%^44^.

### Variables

#### Outcome variables

Primary outcome measures were the self-reported occurrence and severity of the audiovestibular symptoms hearing loss, tinnitus and dizziness in the study population rated on a scale of German language adjectives. Those symptoms were assessed as part of two separate lists of various symptoms in written questionnaires with slightly differing structure. Hearing loss and dizziness were grouped together in one questionnaire while tinnitus was part of the other. Introductory phrases asked to consider each symptom separately and grade it according to severity. The questions were kept identical in both cohorts.

To identify the symptom hearing loss, the German expressions for both “hearing loss” specifically as well as a more general term corresponding to “hearing difficulties” were given. For dizziness, a German term was given that is understood as balance difficulty in its broadest sense, thereby not being specific to vertigo of peripheral vestibular origin. Tinnitus was acquired by initially asking if the symptom occurred at all. A follow-up question allowed for grading if the first answer was positive. With regard to suffering from hearing loss and dizziness, grade 0 meant "not at all", grade 1 "rarely", grade 2 "moderately", and grade 3 "severely". In the case of tinnitus, the occurrence was categorized as grade 0 meaning "no", grade 1 "sometimes", grade 2 "frequently", and grade 3 "always". Grades 2 or 3 of all variables were considered being of clinically relevant severity for subsequent analyses. This was chosen to exclude, as the German phrases for grade 1 suggest, minor or only rarely occurring complaints. This definition of clinical relevance has been applied before in a study on tinnitus on a different subsample from SHIP^46^. The exact wording in German with English translation is given in Supplementary Table S1.

#### Exposure variables

The age at the day of baseline investigation was used for further analyses. Sex was documented in binary categories. Education was assessed during an interview and graded along the German educational framework. Behavioral risk factors included smoking status (never/former or current smoker). Pack years were calculated as average number of cigarettes smoked per day multiplied by the number of years smoking and subsequently divided by 20. Alcohol consumption was assessed in grams ethanol per day derived from a quantity–frequency questionnaire^47^. Physical inactivity was defined as 1 h leisure time of physical activity or less per week.

Body mass index (BMI) and waist-to-hip-ratio were obtained via standardized measurement of body weight and height as well as waist and hip circumference. Diabetes mellitus was defined as insulin resistance with > 8.0 mmol/L non-fasting glucose, reported diagnosis of diabetes, reported medical or reported dietary diabetes treatment. Arterial hypertension was defined as increased systolic or diastolic blood pressure > 140/90 mmHg, reported hypertension or reported antihypertensive medication. Metabolic syndrome was defined using a modification of the approach suggested by Alberti and coworkers as a combination of three out of five parameters: present diabetes mellitus as defined above; abdominal obesity: waist circumference > 94 cm (male) or > 80 cm (female); low HDL-cholesterol: < 1.03 mmol/L (male) or < 1.3 mmol/L (female); high triglycerides: > 2.3 mmol/L non-fasting triglycerides or reported lipid-lowering medication; present hypertension as defined above^48–50^.

For laboratory parameters, random blood samples were collected without stasis from the cubital vein following a standardized protocol, refrigerated to 4–8 °C and shipped on refrigerant packing within 4 to a maximum of 6 h to the laboratory. Serum levels of glucose, total cholesterol, HDL cholesterol, and triglycerides were measured using the Dimension Vista 500 analytical system (Siemens Healthcare Diagnostics, Eschborn, Germany). Dyslipidemia was defined as intake of lipid-lowering medication (EPHMRA ATC code C10) or increased levels of total cholesterol (≥ 6.2 mmol/L), total cholesterol-HDL-cholesterol-ratio (≥ 5.0) or LDL-cholesterol (≥ 4.1 mmol/L).

### Participants

The analyzed population represents a cross-section consisting from the baseline investigations of the cohorts SHIP-START (recruited 1997–2001, baseline investigation designated SHIP-START-0) and SHIP-TREND (recruited 2008–2012, baseline investigation designated SHIP-TREND-0). In total, 8,727 individuals underwent baseline investigations in SHIP-START (n = 4308) and SHIP-TREND (n = 4420). One participant from SHIP-START withdrew consent subsequently. The outcome variables were not completed by 593 participants (SHIP-START n = 93, SHIP-TREND n = 500). Therefore, datasets with the primary variables were available for this analysis from 8134 individuals (93.2%). Details on the population analyzed in this work are given in Supplementary Table S2.

### Analysis and reporting of data

Weighting was used to adjust for bias due to differences in responses, probabilities of selection, as well as discrepancies between data from official statistics and our samples with regard to demographic and geographical distributions^45^. As different methods were used for sampling in SHIP-START and SHIP-TREND, all data except those in Table 1 were standardized using post stratification weighting. The factors included in the post stratification weighting were age, sex, and the data of the local registration office. Based on the data of the non-responder survey, the probability of participation in SHIP-TREND was estimated by means of logistic regression. The resulting inverse probability weights were multiplied by the post stratification weights in SHIP-TREND.

**Table 1:** Difference in weighted prevalence of audiovestibular symptoms between study cohorts by sex.

	Males (n = 4001)	p^†^	Females (n = 4133)	p^†^
Hearing loss		0.001		0.001
*SHIP-START-0*	14.1 (12.5; 15.6)		9.8 (8.5; 11.1)	
*SHIP-TREND-0*	17.9 (16.1; 19.7)		13.5 (11.7; 15.2)	
Tinnitus		0.022		< 0.001
*SHIP-START-0*	8.3 (7.1; 9.5)		7.2 (6.1; 8.4)	
*SHIP-TREND-0*	10.4 (9.0; 11.8)		11.6 (10.0; 13.2)	
Dizziness		0.023		0.011
*SHIP-START-0*	7.8 (6.6; 9.1)		19.8 (18.0; 21.6)	
*SHIP-TREND-0*	10.0 (8.6; 11.5)		16.4 (14.5, 18.2)	
Any symptom		< 0.001		0.177
* SHIP-START-0*	23.1 (21.1; 25.0)		28.6 (26.6; 30.6)	
*SHIP-TREND-0*	28.4 (26.3; 30.5)		30.7 (28.4; 32.9)	

Only symptoms of relevant severity (grade 2 or 3). Any symptom = at least one symptom. Data is reported as weighted prevalence and its 95% confidence interval based on a logistic regression model.

^†^p for difference in prevalence between both studies.

Prevalence of audiovestibular symptoms was reported stratified by sex and age groups. Correlations between the audiovestibular symptoms hearing loss, tinnitus, and dizziness were calculated by phi-coefficients. Characteristics of the study population were stratified by the occurrence of symptoms of relevant severity (grade 2 or 3, i.e. “moderately” or “severely” as well as “frequently” or “always”). Due to non-parametric characteristics of the data, continuous data was reported as median, 25th and 75th percentile and categorical data as percentage.

Group comparisons were conducted by Mann–Whitney U-tests for continuous data and by χ^2^-tests for categorical data. Associations of behavioral and metabolic markers with audiovestibular symptoms were calculated by logistic regression models adjusted for age, sex, and study cohort. To make the odds ratios comparable, continuous exposure variables were standardized before usage in the regression models. In all analyses a p < 0.05 was considered as statistically significant. Analyses were done with Stata 17.0 (Stata Corporation; College Station, USA).

## Results

**Figure 1:**
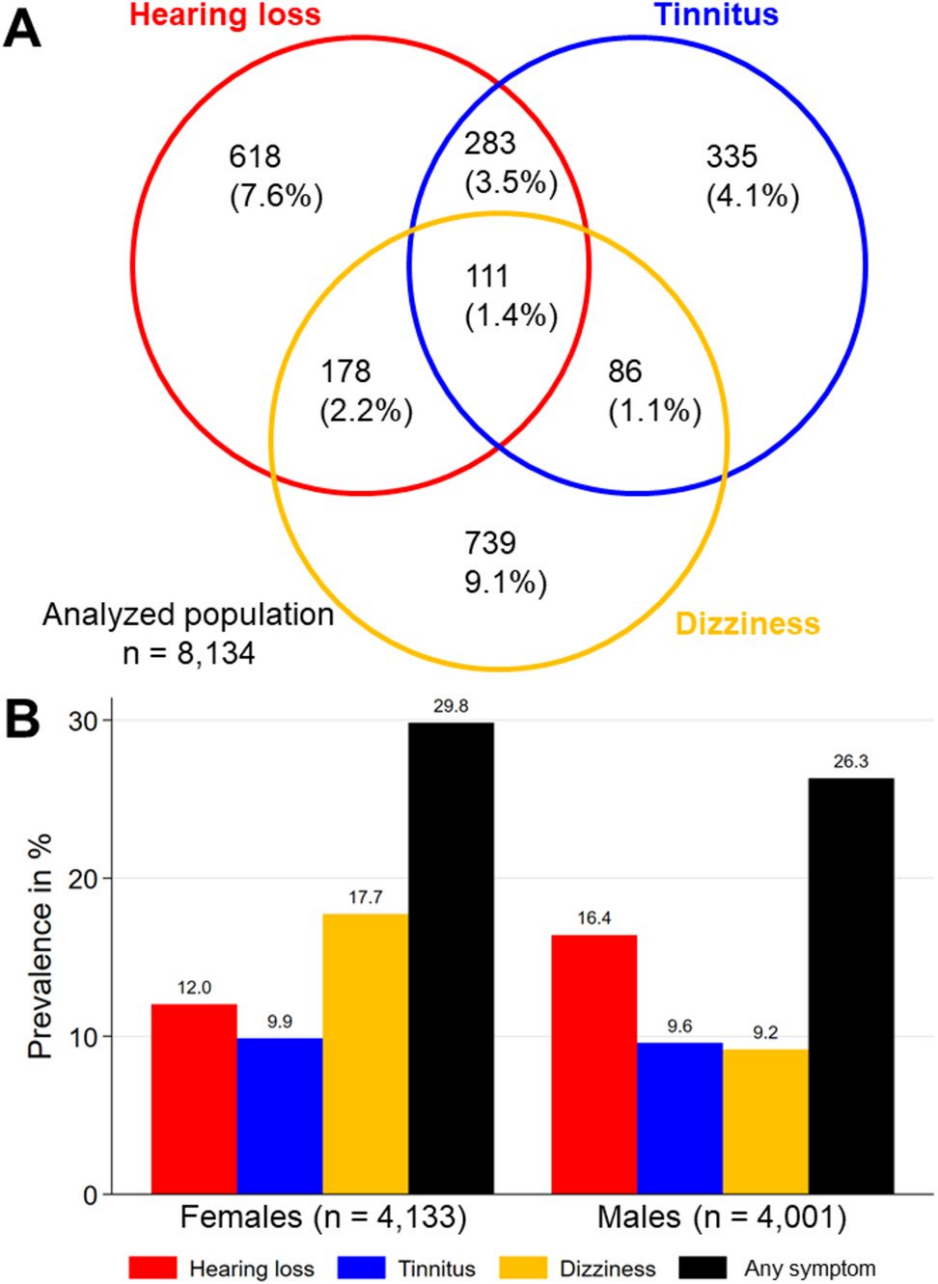
Prevalence and co-prevalence of audiovestibular symptoms in the analyzed population. (**A**) Individual and multiple symptoms (absolute numbers and share). (**B**) Weighted prevalence by sex; any symptom = at least one symptom.

Audiovestibular symptoms of relevant severity (grade 2 or 3) were prevalent in our study population of 8134 individuals with 2350 (28.9%) reporting at least one symptom. Hearing loss affected 1190 individuals (14.6%), tinnitus 815 (10.0%), and dizziness 1114 (13.7%). Notably, 28.0% of cases with symptoms (658/2350) reported more than one (8.1% of total 8134). Among these, 547 (6.7%) had two symptoms, and 111 (1.4%) experienced all three (Fig. 1A). Considering possible sampling bias, we derived a weighted prevalence of 14.2% for hearing loss, 9.7% for tinnitus and 13.5% for dizziness in the Western Pomeranian population. Occurrence of symptoms varied depending on sex, with hearing loss being more prevalent in males and dizziness in females as shown in Fig. 1B.

There was also a trend towards symptoms being associated with each other. The correlation for the co-occurrence of relevant symptoms (phi-coefficient) was 0.318 for hearing loss and tinnitus, 0.127 for hearing loss and dizziness as well as 0.102 for tinnitus and dizziness. This represents a moderate positive relationship between hearing loss and tinnitus that is comparatively stronger than the two other pairings.

A comparison showed differences between the two cohorts that were included in our study. Statistically significant changes in SHIP-TREND-0, that was conducted ten years later than SHIP-START-0, were increased age, education level, body mass index, waist circumference, glucose level in serum, type 2 diabetes, and cholesterol levels. Smoking, alcohol consumption, and rate of arterial hypertension were reduced (Supplementary Table S3). Also, a change in the prevalence of relevant audiovestibular symptoms was noted in SHIP-TREND-0. While hearing loss and tinnitus increased in both sexes in comparison to SHIP-START-0, the prevalence of dizziness showed an increase in males but a decrease in females. In total, the prevalence of any symptom increased in males towards the level of females, where it had not changed significantly (Table 1). Most of the differences between the cohorts disappeared when adjusted for age. In women, however, there remained a statistically significant age-adjusted difference between the cohorts with an increase in tinnitus (SHIP-START-0 7.7% (95% CI 6.4; 8.9), SHIP-TREND-0 11.2% (9.7; 12.7), p < 0.001) and a decrease in dizziness (SHIP-START-0 20.8% (95% CI 18.9; 22.6), SHIP-TREND-0 15.8% (14.1; 17.5), p < 0.001).

To explore potential risk factors, we stratified the entire population according to the absence or presence of any relevant symptoms. While there was no significant difference in the sex ratio regarding the occurrence of symptoms, individuals with symptoms were significantly older. Furthermore, a wide range of lifestyle habits as well as metabolic or cardiovascular risk factors and disorders were significantly associated with the presence of symptoms. An overview is given in Table 2.

**Table 2:** Characteristics of the analyzed population stratified by the occurrence of symptoms.

	No symptom (n = 5784)	Any symptom (n = 2350)	p^†^
Age; years	47 (35; 60)	60 (47; 70)	< 0.001
Females	50.9%	50.5%	0.735
Years of schooling			< 0.001
*< 10 years*	25.1%	44.3%	
*10 years*	51.0%	40.4%	
* > 10 years*	23.9%	15.3%	
Smoking status			< 0.001
*Never*	35.8%	36.5%	
*Former*	32.8%	40.9%	
*Current*	31.4%	22.6%	
Alcohol consumption; g/day	5.05 (1.30; 13.4)	3.59 (0.65; 11.5)	< 0.001
Body mass index; kg/m^2^	26.8 (23.7; 30.2)	27.8 (24.7; 31.0)	< 0.001
Waist circumference; cm	89 (79; 99)	93 (82; 102)	< 0.001
Myocardial infarction	2.1%	5.6%	< 0.001
Stroke	1.4%	4.2%	< 0.001
Glucose; mmol/L	5.3 (4.9; 5.8)	5.4 (5.0; 6.1)	< 0.001
HbA1c; %	5.2 (4.8; 5.6)	5.4 (5.0; 5.9)	< 0.001
Type 2 diabetes	6.9%	13.6%	< 0.001
Total cholesterol; mmol/L	5.3 (4.6; 6.1)	5.4 (4.7; 6.2)	0.024
HDL-cholesterol; mmol/L	1.33 (1.09; 1.60)	1.30 (1.06; 1.58)	0.009
LDL-cholesterol; mmol/L	3.3 (2.6; 3.9)	3.3 (2.7; 4.0)	0.102
Triglycerides; mmol/L	1.40 (0.97; 2.05)	1.54 (1.10; 2.24)	< 0.001
Dyslipidemia	17.6%	30.5%	< 0.001
Arterial hypertension	44.8%	60.0%	< 0.001

Only symptoms of relevant severity (grade 2 or 3). Any symptom = at least one symptom. Continuous data are expressed as median, 25th and 75th percentile; categorical data as percentage.

^†^Mann–Whitney-U test (continuous data) or χ^2^-test (categorical data).

**Figure 2:**
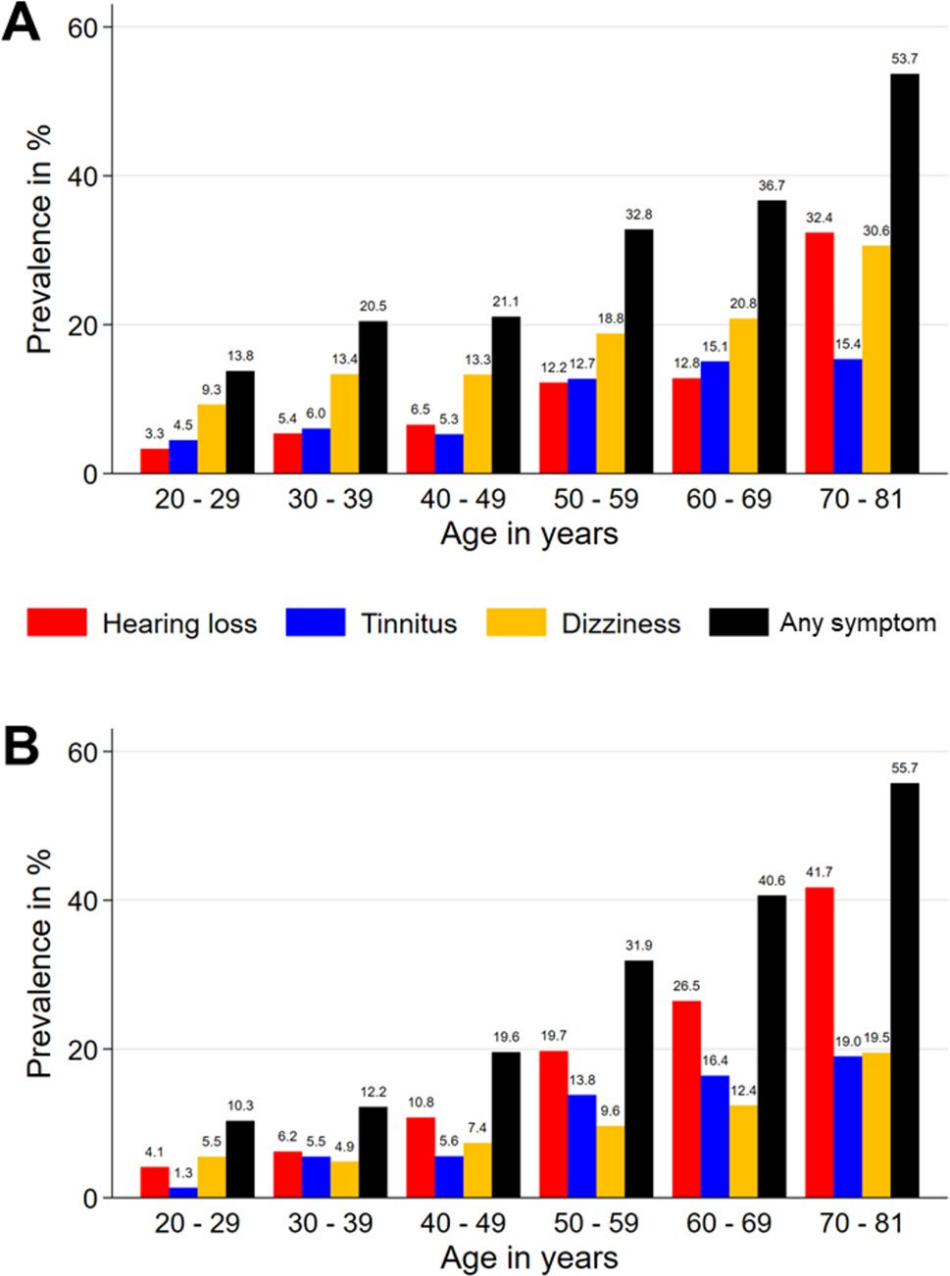
Prevalence of audiovestibular symptoms by age group and sex. (**A**) Female, (**B**) male. Any symptom = at least one symptom.

In addition, the presence of relevant single or combined audiovestibular symptoms increased markedly with age (Fig. 2). Among 2614 individuals aged 60 or older, 1170 (44.8%) reported at least one, 391 (15.0%) two and 69 (2.6%) all three symptoms. This was even more pronounced among 1097 individuals aged 70 or older with 579 (52.8%) reporting at least one, 218 (19.9%) two and 38 (3.5%) all three. The same trend was observed when weighted prevalence was broken down by age and sex. In the oldest age group of 70–81 years at the day of investigation, the majority of individuals reported at least one symptom in both sexes. Dizziness was the most dominant symptom in females until being overtaken by hearing loss in the oldest age group (Fig. 2A). In contrast, the dominant symptom in males was hearing loss from the age group of 30–39 on, increasing from a weighted prevalence of 6.2% to 41.7% in the oldest group (Fig. 2B).

Following this, further analyses were adjusted for age and sex (Table 3). Education emerged as a prominent factor, significantly reducing the odds of experiencing any relevant symptoms by 45% for individuals with more than 10 years of schooling compared to those with less than 10 years. This effect was most pronounced for dizziness, followed by hearing loss. Smoking was the only factor that was significantly associated with all three audiovestibular symptoms as well as the only risk factor significantly associated with tinnitus at all. Notably, this association was most distinct when comparing former smokers to individuals who never smoked. The presence of type 2 diabetes and higher triglycerides showed a statistically significant association with the occurrence of both hearing loss and dizziness. Elevated levels of serum glucose and the presence of arterial hypertension were significantly associated with increased odds of hearing loss. On the other hand, HbA1c, serum HDL-cholesterol, and a diagnosis of dyslipidemia were positively associated with dizziness only.

**Table 3:** Association of behavioural and metabolic factors with audiovestibular symptoms.

	Hearing loss (n = 1190)	Tinnitus (n = 815)	Dizziness (n = 1114)	Any symptom (n = 2350)
	Odds ratio (95% CI)	Odds ratio (95% CI)	Odds ratio (95% CI)	Odds ratio (95% CI)
Education (years of schooling)				
*10 years vs. < 10 years*	0.85 (0.71; 1.03)	0.93 (0.75; 1.15)	0.76 (0.63; 0.92)*	0.82 (0.71; 0.95)*
*> 10 years vs. < 10 years*	0.57 (0.46; 0.72)*	0.79 (0.61; 1.01)	0.48 (0.38; 0.62)*	0.55 (0.46; 0.65)*
Smoking status				
*Former vs. never*	1.27 (1.06; 1.52)*	1.24 (1.01; 1.53)*	1.24 (1.04; 1.48)*	1.30 (1.13; 1.48)*
*Current vs. never*	1.22 (0.99; 1.50)	1.09 (0.87; 1.37)	1.05 (0.87; 1.29)	1.19 (1.03; 1.38)*
Alcohol consumption; g/day	1.06 (0.98; 1.14)	1.03 (0.95; 1.12)	0.89 (0.78; 1.01)	1.01 (0.95; 1.08)
Body mass index; kg/m^2^	1.03 (0.96; 1.11)	1.00 (0.92; 1.09)	1.04 (0.97; 1.12)	1.00 (0.94; 1.06)
Waist circumference; cm	1.08 (0.99; 1.17)	1.02 (0.92; 1.12)	1.07 (0.98; 1.17)	1.02 (0.96; 1.10)
Glucose; mmol/L	1.07 (1.00; 1.14)*	1.05 (0.96; 1.14)	1.05 (0.97; 1.13)	1.05 (0.99; 1.11)
HbA1c; %	1.07 (0.99; 1.15)	1.03 (0.94; 1.13)	1.10 (1.02; 1.18)*	1.08 (1.02; 1.15)*
Type 2 diabetes	1.37 (1.10; 1.72)*	1.14 (0.86; 1.49)	1.48 (1.16; 1.88)*	1.35 (1.12; 1.64)*
Total cholesterol; mmol/L	0.98 (0.90; 1.06)	1.03 (0.95; 1.13)	0.94 (0.86; 1.02)	0.96 (0.90; 1.01)
HDL-cholesterol; mmol/L	0.98 (0.89; 1.07)	0.97 (0.89; 1.07)	0.91 (0.84; 0.99)*	0.97 (0.91; 1.04)
LDL-cholesterol; mmol/L	0.95 (0.88; 1.03)	1.04 (0.95; 1.13)	0.95 (0.88; 1.03)	0.94 (0.88; 0.99)*
Triglycerides; mmol/L	1.11 (1.02; 1.20)*	1.03 (0.95; 1.11)	1.09 (1.01; 1.18)*	1.09 (1.02; 1.16)*
Dyslipidemia	1.17 (0.99; 1.39)	1.22 (0.99; 1.49)	1.46 (1.22; 1.75)*	1.33 (1.16; 1.53)*
Arterial hypertension	1.23 (1.04; 1.45)*	1.04 (0.86; 1.25)	1.12 (0.94; 1.32)	1.13 (0.99; 1.28)

Only symptoms of relevant severity (grade 2 or 3). Any symptom = at least one symptom. Data are derived from logistic regression models adjusted for age and gender. The exposure variables were included separately and not together into the models. Continuous exposure were standardized. *CI* confidence interval.

*p < 0.05.

**Figure 3:**
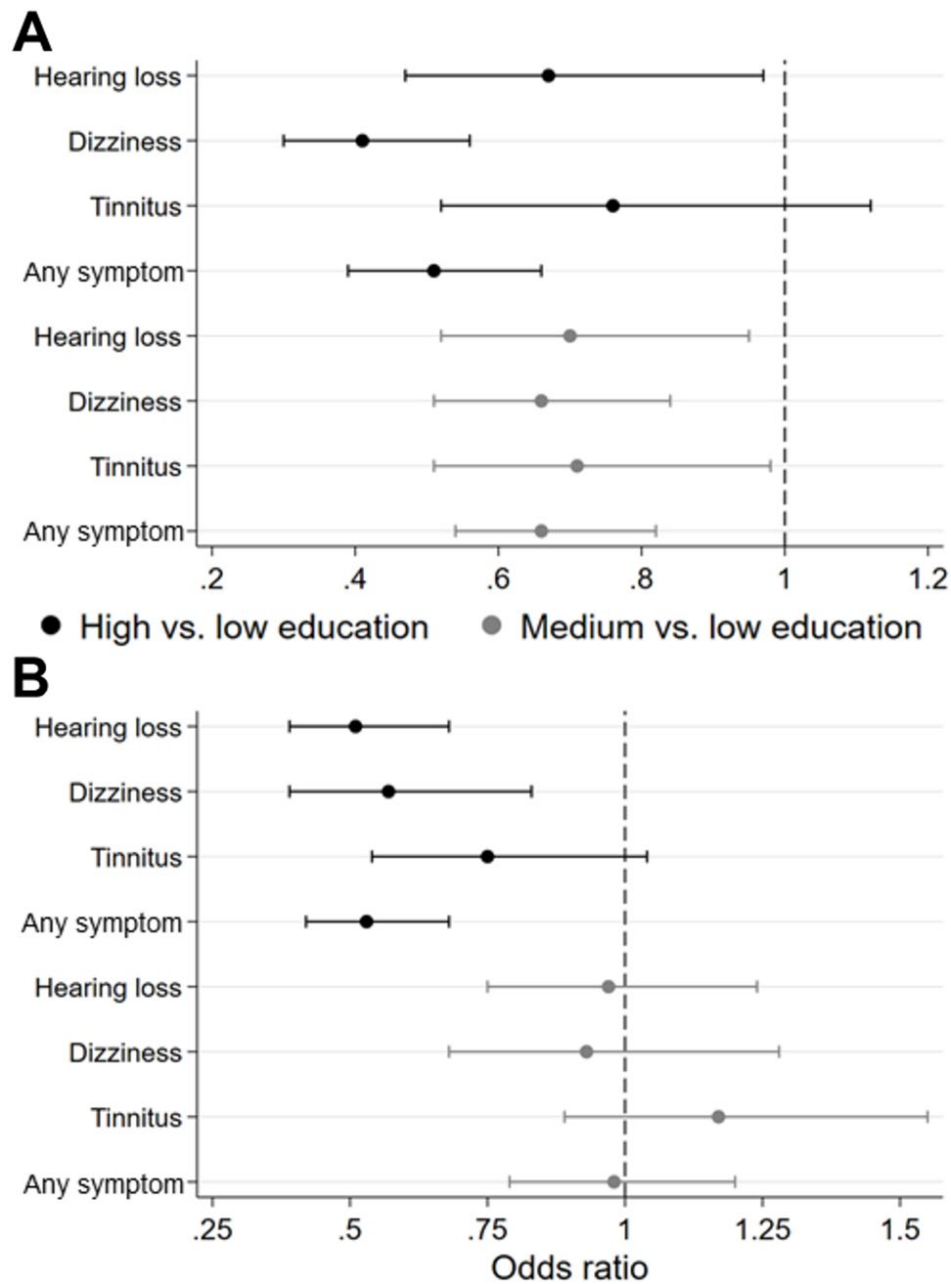
Age-adjusted associations of education with audiovestibular symptoms stratified by sex. (**A**) Female, (**B**) male. Odds ratio and 95% confidence interval. Only symptoms of relevant severity (grade 2 or 3). Any symptom = at least one symptom.

**Figure 4:**
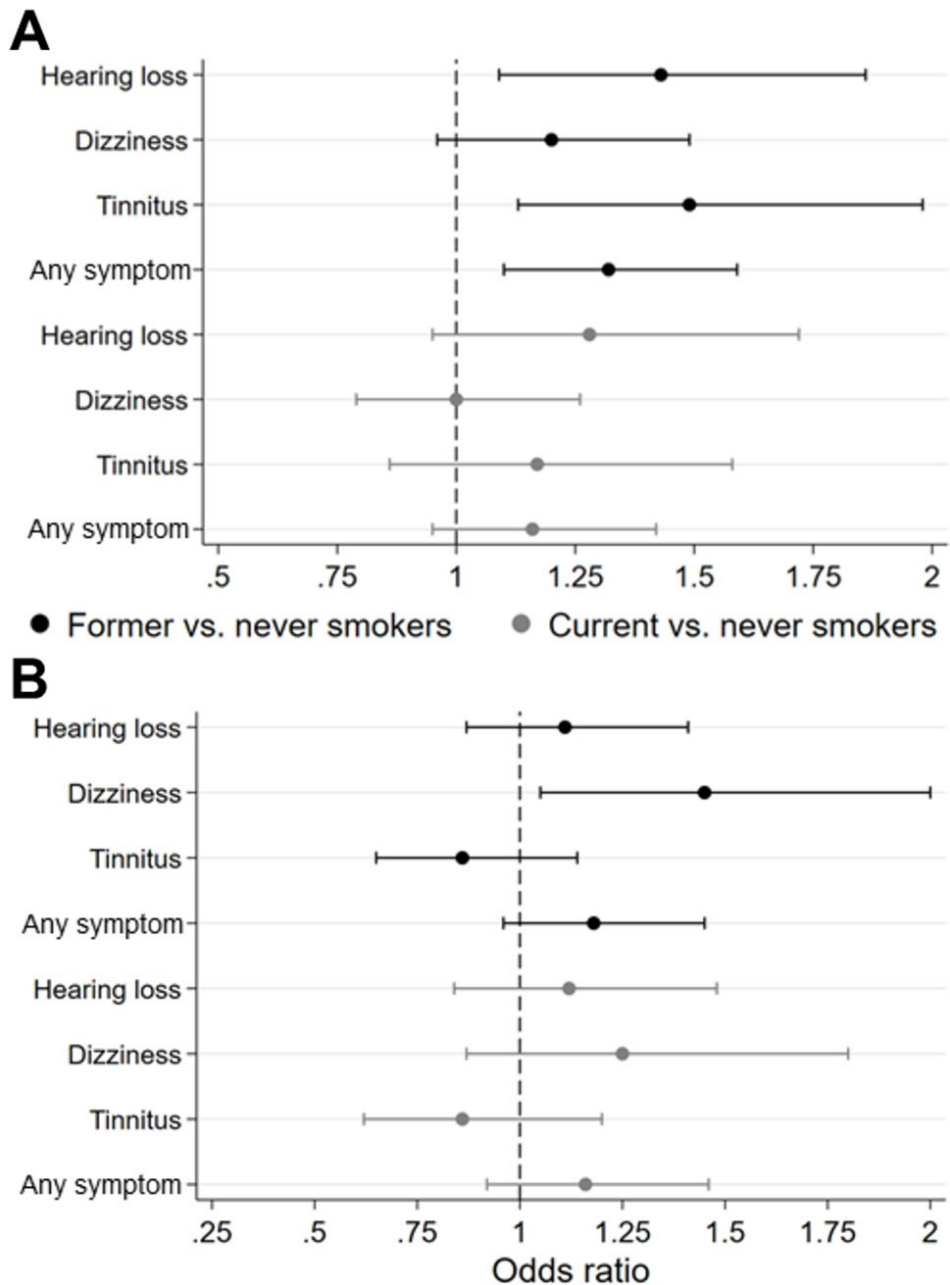
Age-adjusted associations of smoking status with audiovestibular symptoms stratified by sex. (**A**) Female, (**B**) male. Odds ratio and 95% confidence interval. Only symptoms of relevant severity (grade 2 or 3). Any symptom = at least one symptom.

Subsequently, an in-depth exploration of the factors of education and smoking was conducted to assess their relevance for single symptoms and the occurrence of any symptom, broken down by sex. Higher education was associated with reduced odds for hearing loss, dizziness, tinnitus, and any symptom in both sexes. Additionally, medium education, as compared to low education, significantly decreased the odds for all individual symptoms in both sexes as well as for any symptom in females only. The most notable odds ratios were observed for high versus low education, indicating reduced odds for dizziness in females of 0.45 (95% CI 0.30; 0.56) and hearing loss in males of 0.51 (95% CI 0.39; 0.68) as well as any symptoms in females of 0.51 (95% CI 0.39; 0.66) and males of 0.53 (95% CI 0.42; 0.68), respectively (Fig. 3). Former female smokers showed significantly increased odds for hearing loss, tinnitus, and any symptom while in males, this association was only observed for dizziness. No significant findings were identified regarding current versus never smokers in both sexes (Fig. 4).

## Discussion

Our results established a high prevalence and co-prevalence of self-reported audiovestibular symptoms in a population-based sample from rural Western Pomerania. It is important to note that each symptom has a different, but considerably overlapping set of possible causes arising from a wide range of topographic locations of the ear or associated systems.

Assessing the prevalence of hearing loss, tinnitus, and dizziness is challenging. The reason for this is a wide variety of available methods and additionally, the three symptoms differ substantially in this regard. Hearing loss can be assessed by self-report via interview or structured questionnaires or by audiometric diagnostic tests, like pure tone audiograms. For tinnitus and dizziness, prevalence estimates are mainly based on self-report since there is even less consensus on a single objective measurement method or internationally standardized recording model for tinnitus^51^ or dizziness^52^. Self-report as an outcome measure has advantages, since it is very cost-effective and can be seamlessly included into any structured interview. For hearing loss, the validity in comparison to established diagnostic tests has been investigated in great detail. The value of a single screening question in comparison to audiometry is well described and reaches a sensitivity and specificity of 80% and 74%, respectively^53^. Likewise, in epidemiological studies, self-reported hearing loss was validated as an estimate for audiometrically determined hearing loss: Sindhusake and coworkers summarized different short questions on subjective hearing loss that yielded a sensitivity of 66–78% and a specificity of 67–80% for audiometrically proven mild hearing loss. For moderate hearing loss, the sensitivity was given as 90–93% and the specificity as 56–71%^54^. Therefore, since self-reported moderate to severe hearing loss correlates with audiometric diagnostic tests with high sensitivity, this method can be applied to gain insight into the prevalence and impact of hearing difficulties in large population-based studies. A drawback of our data in this regard is, however, that our questionnaires were not validated with regard to audiometry. Additionally, we analyzed data that was collected over the long timespan from 1997 to 2012. Considering also relevant societal transformation in East Germany during this period, this might contribute to heterogeneity in our data. However, the choice of the baseline investigations of the available cohorts allowed us to achieve a maximum number of individuals for the present study.

The pathology behind hearing loss, tinnitus and dizziness in general varies a lot and no single diagnostic tests can fully capture the complaints as perceived by an individual. Self-reported measures may be able to assess impairments beyond middle or inner ear dysfunction. For the generation of datasets sufficiently large for genome-wide association studies, self-reported hearing loss is even considered as a distinct auditory phenotype^55^. While acknowledging the limitations of self-reported symptoms in the absence of diagnostic tests, self-report does capture the whole and thereby complex processing pathway from basic sensory perception to cognition, including emotional assessment and mood as well as cardiovascular and neurological status. Moreover, societal factors play a role. Self-reported measures can be modified by increased awareness or higher health expectations. That has been brought forward as an explanation of an age-specific increase in the prevalence of tinnitus in age groups that were five years apart despite an overall declining prevalence of hearing loss in a study from Wisconsin, USA^56^. However, major limitations of self-reported measures have been described that should be considered in interpreting our results. Again, this has been described most extensively for hearing loss, where discrepancies between self-report and audiometry have been reported for a long time. In an earlier study with considerably comprehensive characterization of hearing phenotype, 20.2% of the subjects with self-reported hearing loss did not even meet the threshold of mild hearing loss in a pure-tone audiogram while 6.2% of the individuals reporting no hearing problem did have at least mild hearing loss in audiometry^57^. Specific risks of misreporting depending on population properties may even limit the validity and generalizability of self-report to a varying degree in subpopulations. Individual risk factors or the frequency range of hearing loss may also have an effect. Discrepancies like over- or underreporting of audiometrically determined hearing loss can result from exposure to occupational noise as well as hypotension and depression^58^. In middle-aged and older adults, self-reported measures of hearing had limited accuracy. They were also not sufficiently sensitive to detect hearing loss at 1 kHz and 3 kHz when compared to a hearing screening device. Factors associated with misreport were sex, age, education and unfavorable lifestyle habits^59^. Other limitations have been described in occupational groups with noise exposure, showing varying performance characteristics, in particular in high frequencies^60^, thereby underlining the relevance of audiometry as a refence. In older adults, trends toward underestimation of hearing loss may systematically lead to consecutive underestimation of the magnitude of associations of hearing impairment with functional outcomes^61^. Those considerations are of particular importance with regard to our study, where age was a major determinant.

For tinnitus, a subjective sensation of the auditory pathway by definition, no objective diagnostic test exists^51^. Thus, self-report is the best available measure for tinnitus in epidemiological studies and usually presents a point prevalence. In previous studies, the definition of tinnitus and consequently the phrasing of questions for the assessment of tinnitus varied and limited the conclusions that could be drawn from meta-analyses^2,62^. While the question used in our study allowed for the grading of severity, it did not include the duration or period of the symptom. The inclusion of a minimum duration time like “more than five minutes”, may have yielded a more focused response. Assessing balance disorders, simple or structured questions are considered to have unsatisfactory sensitivity and specificity for pathological findings in vestibular testing. This has previously been demonstrated by the poor predictive capability of a widely used questionnaire in epidemiological research^63^. The phrasing of questions for self-report of dizziness is complex and linguistically nuanced. The German term ‘Schwindel’ does not clearly distinguish between ‘dizziness’ and ‘vertigo’ but can include a wide variety of sensations. It  does also not allow any conclusions to be drawn about the genesis. The distinction between ‘dizziness’ and ‘vertigo’ has been debated, with the latter presumed to be more specific for a rotational perception indicative of peripheral vestibular disorders^3^. Accordingly, secondary data such as health insurance registers for dizziness are also subject to bias. In primary care in particular, a plausible diagnosis can only be found in a minority of cases for patients who present with dizziness as the main symptom. For a considerable share of cases, this applies even after extended diagnostics^64,65^. Thereby, despite progress in defining these terms^52^, there remains no universally accepted solution for epidemiological investigations.

Basic properties as well as prevalence shifted slightly between the cohorts in our analyzed population, with the median age of SHIP-START-0 being 50 (range 36; 64 for 25th and 75th percentile) and of SHIP-TREND-0 being 52 (39; 63). Therefore, to avoid confounding between the two cohorts, adjustments were made for the slightly different age and sex distribution in SHIP-START and SHIP-TREND. Nevertheless, we observed an increase in the prevalence of hearing loss and tinnitus in both sexes and an increase in dizziness in men between the two cohorts. These shifts within a comparable short timeframe of about 10 years, separating SHIP-START and SHIP-TREND cohorts, run counter to a possible decrease due to social development, health-related legislation or public-health programs. Despite these unexpected trends in our study, relevant changes in prevalence have already been reported for thyroid disorders^66^ and lifestyle-related risk factors^67^ in our population. The observed changes in audiovestibular health within the population raise interesting questions and therefore warrant further investigation.

Our results indicate a high prevalence of hearing disorders in the Western Pomeranian population. In population-based studies that included all age ranges of adult individuals, the prevalence of self-reported hearing loss ranged from 13.0 to 26.8% in studies from Eastern Asia and Northern America^26,58^, placing our result within this range of developed countries with high life expectancy. This is, however, considerably higher than a worldwide estimate that places the prevalence of moderate to severe hearing loss at 5.2% globally and 5.8% for the European region, where the present study originated. This estimate notably excluded self-reported hearing loss and thereby only considered prevalence figures based on audiological assessment^68^. A German study extrapolated a prevalence of 16.2% for mild and 5.8% for moderate hearing loss for the national population based on two different investigations in three samples based on audiometry^69^. For tinnitus, we observed a weighted prevalence of 9.7% that is within the lower range of previous reports. Prevalence values from 5.1% to 42.7% have been given before when tinnitus was assessed without specific qualifier and 3.0% to 30.9% when only bothersome tinnitus was considered^2^.

With an equally large population-based cohort, Neuhauser and coworkers identified a much higher prevalence of 29.5% for self-reported moderate or severe dizziness compared to 13.5% in our study. Of note, in a consecutive focused neurotological interview, only 24.2% of those individuals reported symptoms that were suggestive of a peripheral vestibular disorder^41^. With their similarly epidemiologically representative cohort, it is likely that a comparable distribution regarding peripheral origin underlies the dizziness complaints determined in our population. Other estimates for the prevalence of dizziness without consideration of a potential peripheral vestibular origin range from 15% to 35%^3,70^, again placing our result in the lower range. When vertigo is considered separately as dysfunction of the peripheral vestibular organ in the inner ear, 3% to 10% are given^3^. However, in a community-based study from north-eastern France, the view of ‘vertigo’ as a term for primarily vestibular dysfunction is challenged. The reported one-year prevalence of vertigo was as high as 48.3%, while unsteadiness and dizziness were noted by 39.1% and 35.6% of individuals, respectively^71^.

Although audiovestibular symptoms are assumed to be common, the variance in prevalence numbers in the literature can most likely be attributed to differences in the definition of disorders, cut-off values in diagnostic tests, and various assessment methods^2,3,72^. Additionally, prevalence can be assumed to be heavily influenced by factors like age, as determined by the age distribution in the underlying population. Likewise, studies on older populations typically yield a higher prevalence of symptoms. Furthermore, point prevalence, period prevalence and lifetime prevalence are often not clearly distinguishable by self-report. In the Framingham cohort (age 60 and older), self-reported hearing loss was noted in 41.1%, mild hearing loss in 29%, and tinnitus in 16.8% of individuals^57^. Similarly, the Blue Mountains Hearing Study (age 49 and older) reported prevalence rates of 39.7%, 13.9%, and 2.3% for mild, moderate, and severe self-reported hearing loss, respectively^54^. Among the individuals investigated here, hearing loss was more prevalent in males while dizziness dominated in females. Beyond this, prevalence rose markedly with advancing age, leading to the majority of individuals at 70 years or older reporting audiovestibular symptoms. The leading contributor here was hearing loss in both sexes, closely followed by dizziness in females. While age-related hearing loss and vestibular impairment have been investigated separately in most studies, there is ample evidence that there are underlying similarities in the mechanisms leading to both forms of sensory deficiency^21^.

Beyond prevalence, we studied the association of individual risk and lifestyle-related factors with audiovestibular symptoms. Education was the most prominent factor in our analyses, significantly reducing the odds of any relevant symptom by 45% as well as hearing loss and dizziness individually when more than 10 years of schooling were compared to less than 10 years. This is an interesting finding, since cognitive stimulation, approximated by the level of education attained during life and strongly influenced by hearing and communication abilities, is also a major factor influencing dementia risk in later life^73^. Furthermore, a strong association is assumed between hearing loss and cognitive decline^74^. The process of neurodegeneration is supposed to be the underlying pathomechanism of central and peripheral auditory dysfunction. Simultaneously, cognitive decline may be signified early by hearing difficulties and reinforced by sensory deprivation^75^. Sufficient hearing rehabilitation may even be able to abrogate the effect of hearing loss on the development of dementia^76^. A risk of dementia was reported to be further increased when multiple sensory modalities are impaired^77^, further hinting a close connection between sensory loss and neurodegeneration and possibly a shared pathway of neurodegeneration affecting both cognition and sensory systems at once. Additionally, increasing education in conjunction with reduced occupational noise exposure, ear infections and smoking in the population are considered to contribute to a decreased incidence of ear disorders, especially hearing loss^34,78^.

By the investigation of cardiovascular risk factors, associations of audiovestibular symptoms with smoking, diabetes, dyslipidemia and hypertension were found. Smoking was the only single factor that was significantly associated with all three audiovestibular symptoms in our study. In the past, similar associations for smoking and decreased audiovestibular health have been reported mostly individually for hearing loss^30,32,34–37^, tinnitus^9,33,39^, and dizziness^40,41^. In an animal model of oxidative stress, degeneration and loss of cochlear spiral ganglion neurons have been documented after chronic exposure to cigarette smoke^79^. This mechanism might be equally relevant for the development of tinnitus or dizziness, as other neuronal tissues are also degraded. In previous reports, diabetes mellitus was associated with dysfunction of the auditory and vestibular systems^9,32,40,80,81^. This has been described in relation to hearing loss^32,37,80,81^, balance disturbance^40^ as well as tinnitus^9^. In our data, however, diabetes was linked to hearing loss and dizziness but not to tinnitus. The complex relationship between diabetes mellitus and hearing loss has been explored in the past and likely includes a multitude of different routes like toxic effects of hyperglycemia, diabetic microangiopathy and neuropathy as well as side-effects of diabetic medications^81^. Most likely it already takes place at a precursor stage of diabetes^82^. Additionally, for the relationship between diabetes and balance dysfunction, a possible role for central insulin resistance has been suggested^83^. The association found here between dyslipidemia as well as hearing loss and dizziness confirms earlier reports in this regard^41,84^, but a detailed mechanism is still warranted. While in a mouse model of hyperlipidemia, the development of hearing loss could be prevented by a statin^85^, a cross-sectional study on humans did not find dyslipidemia alone to be directly associated with hearing loss^86^. A diagnosis of arterial hypertension was significantly associated with hearing loss in our data, confirming earlier studies^32,37^. It was, however, not associated with tinnitus or dizziness. Previous reports linked this condition to tinnitus^9^ and dizziness^40,41,84^ as well. Looking at our findings on cardiovascular risk factors and audiovestibular dysfunction in light of the body of evidence from the literature, a strong but complex link can be assumed. However, due to a multifaceted interaction, the effect sizes may be too small to be assessed in detail by surrogate markers.

An interaction between function and dysfunction of the senses of hearing and balance is widely assumed. Through the calculation of the phi-coefficient, we identified a moderate positive relationship between hearing loss and tinnitus with inconclusive results for the other two pairings. Another population-based cross-sectional study in 2,751 individuals of 50 years and older found a significant association between dizziness and tinnitus, but not between dizziness and hearing loss^87^. In a clinical setting, age-related high frequency-hearing loss was not associated to measurable peripheral vestibular dysfunction at the unaffected side in 185 adult patients with unilateral cerebello-pontine angle tumors^24^, emphasizing that the detailed mechanism of interaction between auditory and vestibular dysfunction is still elusive. In comparisons of genome-wide association studies, dizziness has been reported moderately correlated with hearing loss as well as tinnitus. To investigate this, phenotypes of dizziness have been studied in the Million Veterans Program and in a meta-analysis of cohorts from Iceland, Finland, the UK Biobank as well as the USA while hearing loss and tinnitus were explored in the UK Biobank^88^. It is still necessary to gain more insight in the future, since a detailed mechanistic understanding is lacking. Of particular importance is a reported strong association of multisensory impairment with neurodegeneration, an association that has been already found for vision, hearing, smell, and touch^77^ and could be applicable to the interaction of hearing and balance as well.

## Conclusion

The study revealed a high prevalence of audiovestibular symptoms in the Western Pomeranian population. A relevant subgroup reported more than one symptom, thus highlighting the multilayered relations between hearing loss, tinnitus, and dizziness, which have been assessed only separately in previous studies.

Sex and age have a major influence on the occurrence of audiovestibular symptoms. The prevalence of hearing loss and tinnitus increased significantly over a period of approximately 10 years when our two population-based cohorts SHIP-START and SHIP-TREND were compared. The study identifies education as a protective factor against audiovestibular symptoms, while from all factors studied, only smoking was associated with the occurrence of all three audiovestibular symptoms at once. The risk for hearing loss only was increased by higher levels of glucose and arterial hypertension while dizziness only was facilitated by dyslipidemia as well as increased levels of HbA1c. These findings contribute to our understanding of the complex interplay between lifestyle and health outcomes.

Further studies utilizing more refined assessment instruments and longitudinal methodologies are essential to elucidate the intricate interplay among audiovestibular symptoms. Furthermore, a deeper understanding of the mechanisms involved in risk and lifestyle factors necessitates more detailed exploration. Broad cohort studies on the prevalence of audiovestibular symptoms are essential for public health planning, improving clinical care, understanding epidemiological patterns, identifying risk factors, adopting a holistic health approach, and guiding future research initiatives in the field of auditory and vestibular health.

### Supplementary Information

Supplementary Tables.

## Data Availability

The data that support the findings of this study are not openly available due to national data protection laws. However, they can be obtained individually from the corresponding author upon reasonable request. Data are located in controlled access data storage at University Medicine Greifswald (https://transfer.ship-med.uni-greifswald.de/).
