# Supplementary material for: Prevalence and risk factors of self-reported hearing loss, tinnitus, and dizziness in a population-based sample from rural northeastern Germany
Source: Sci Rep. 2024 Jul 31;14:17739. doi: 10.1038/s41598-024-68577-3 (PMC11291685; doi:10.1038/s41598-024-68577-3)
Supplement: Supplementary file 1 — Supplementary Tables. [file 41598_2024_68577_MOESM1_ESM.docx]

# Supplementary Information

for the article

“Prevalence and risk factors of self-reported hearing loss, tinnitus, and dizziness in a population-based sample from rural northeastern Germany”

by

Friedrich Ihler^1^, Tina Brzoska^1^, Reyhan Altindal^1^, Oliver Dziemba^1^, Henry Völzke^2^, Chia-Jung Busch^1^, Till Ittermann^2^

1: Department of Otorhinolaryngology, Head and Neck Surgery, University Medicine Greifswald, 17475 Greifswald, Germany

2: Institute for Community Medicine, University Medicine Greifswald, 17475 Greifswald, Germany

## Supplementary Table S1: Phrasing and grading of primary outcome measures.

|  | **Questions** | **Options** | | | |
| --- | --- | --- | --- | --- | --- |
| **Introductory remarks for complaints that include hearing loss and dizziness.**  ***(English translation)*** | Im Folgenden wird eine Reihe von Beschwerden genannt. Machen Sie bitte bei jeder aufgeführten Beschwerde ein Kreuz in eines der vier Kästchen, je nachdem, ob sie gar nicht, kaum, mäßig oder stark unter diesen Beschwerden leiden.  *In the following several of disorders will be named. Please give an answer to every below-mentioned disorder and decide where to tick depending on how strong you suffer from these symptoms.* |  | | | |
| **Hearing loss**  ***(English translation)*** | Schwerhörigkeit, Hörbeschwerden  *Hearing loss, hearing difficulties* | Gar nicht *Not at all* | Kaum  *Rarely* | Mäßig  *Moderately* | Stark  *Severely* |
| **Dizziness**  ***(English translation)*** | Schwindelgefühl  *Dizziness* | Gar nicht *Not at all* | Kaum  *Rarely* | Mäßig  *Moderately* | Stark  *Severely* |
| **Introductory remarks for complaints that include tinnitus**  ***(English translation)*** | Leiden Sie an den im folgenden genannten Beschwerden?  *Do you suffer from one of the following complaints?* |  | | | |
| **Tinnitus occurrence**  ***(English translation)*** | Ohrgeräusche, Ohrensausen  *Tinnitus/ ringing in the ears* | Nein  *No* | Ja  *Yes* | | |
| **Tinnitus severity**  ***(English translation)*** | Wenn ja, wie häufig?  *If yes, how often?* |  | Manchmal  *Sometimes* | Häufig  *Frequently* | Immer  *Always* |
| **Severity grade** |  | 0 | 1 | 2 | 3 |
| The highlighted severity grades 2 and 3 were considered as clinically relevant values for the analysis in this study. | | | | | |

## Supplementary Table S2: Excluded participants and analyzed population.

|  | **Excluded from analysis**  **(n = 593)** | **Analyzed population**  **(n = 8,134)** | **p^†^** |
| --- | --- | --- | --- |
| **Females** | 56.3% | 50.8% | 0.009 |
| **Age; years** | 62 (46; 73) | 51 (38; 63) | < 0.001 |
| **Age groups; years**  **20-30**  **30-40**  **40-50**  **50-60**  **60-70**  **70-80** | 4.9%  13.5%  11.5%  15.7%  19.9%  34.6% | 11.1%  17.0%  19.3%  20.0%  18.8%  13.9% | < 0.001 |
| **Education**  **< 10 years**  **= 10 years**  **> 10 years** | 45.2%  42.8%  12.0% | 30.6%  47.9%  21.5% | < 0.001 |
| **Smoking**  **Current smoker**  **Former smoker**  **Never smoker** | 38.1%  36.9%  25.0% | 36.0%  35.2%  28.8% | 0.155 |
| **Alcohol consumption; g/day** | 1.5 (0.0; 6.5) | 4.6 (1.1; 13.1) | < 0.001 |
| **Body mass index; kg/m²**  **Waist circumference; cm** | 28.6 (25.3; 32.3)  94 (84; 105) | 27.1 (24.0; 30.5)  90 (79; 100) | < 0.001  < 0.001 |
| **Diabetes mellitus** | 16.1% | 8.8% | < 0.001 |
| **Hypertension** | 65.6% | 49.2% | < 0.001 |
| **Dyslipidemia** | 25.7% | 21.4% | 0.016 |
| Continuous data are expressed as median, 25th and 75th percentile; categorical data as percentage;  ^†^: Mann-Whitney-U test (continuous data) or χ2-test (categorical data). | | | |

## Supplementary Table S3. Characteristics of the analyzed population stratified by study cohort.

|  | **SHIP-START-0** | **SHIP-TREND-0** | **p^†^** |
| --- | --- | --- | --- |
| **N** | 4,215 (51.8%) | 3,919 (48.2%) |  |
| **Age in years** | 50 (36; 64) | 52 (39; 63) | 0.007 |
| **Sex** |  |  |  |
| Female | 2,140 (50.8%) | 1,993 (50.8%) | 0.949 |
| Male | 2,075 (49.2%) | 1,927 (49.2%) |  |
| **Education** |  |  |  |
| < 10 years | 1,670 (39.8%) | 813 (20.8%) | < 0.001 |
| = 10 years | 1,834 (43.7%) | 2,053 (52.5%) |  |
| > 10 years | 694 (16.5%) | 1,046 (26.7%) |  |
| **Smoking status** |  |  |  |
| Never smokers | 1,504 (35.7%) | 1,421 (36.3%) | 0.005 |
| Former smokers | 1,429 (34.0%) | 1,425 (36.4%) |  |
| Current smokers | 1,276 (30.3%) | 1,065 (27.2%) |  |
| **Alcohol consumption in g/d** | 5.26 (1.30; 14.92) | 3.62 (0.72; 10.96) | < 0.001 |
| **Body mass index in kg/m^2^** | 26.84 (23.72; 30.07) | 27.40 (24.38; 30.86) | < 0.001 |
| **Waist circumference in cm** | 89.3 (78.8; 99.0) | 90.0 (80.0; 100.5) | < 0.001 |
| **Myocardial infarction** | 141 (3.4%) | 109 (2.8%) | 0.136 |
| **Stroke** | 93 (2.2%) | 84 (2.1%) | 0.839 |
| **Glucose in mmol/L** | 5.3 (4.9; 5.8) | 5.4 (4.9; 5.9) | < 0.001 |
| **HbA1c in %** | 5.3 (4.9; 5.8) | 5.2 (4.9; 5.6) | < 0.001 |
| **Self-reported type 2 diabetes** | 335 (8.0%) | 383 (9.8%) | 0.004 |
| **Total cholesterol in mmol/L** | 5.3 (4.6; 6.1) | 5.4 (4.6; 6.1) | 0.036 |
| **HDL-Cholesterol in mmol/L** | 1.24 (1.00; 1.52) | 1.39 (1.16; 1.66) | < 0.001 |
| **LDL-Cholesterol in mmol/L** | 3.24 (2.62; 3.86) | 3.30 (2.67; 3.95) | 0.013 |
| **Triglycerides in mmol/L** | 1.55 (1.08; 2.29) | 1.34 (0.94; 1.95) | < 0.001 |
| **Self-reported dyslipidemia** | 834 (21.9%) | 794 (20.8%) | 0.256 |
| **Arterial hypertension** | 2,196 (52.2%) | 1,798 (46.0%) | < 0.001 |
| Continuous data are expressed as median, 25th and 75th percentile; categorical data as percentage;  ^†^: Mann-Whitney-U test (continuous data) or χ2-test (categorical data) | | | |
